# Supplementary figures and images for: Comparative analysis of prophage-like elements in Helicobacter sp. genomes
Source: PeerJ. 2016 May 5;4:e2012. doi: 10.7717/peerj.2012 (PMC4860318; doi:10.7717/peerj.2012)

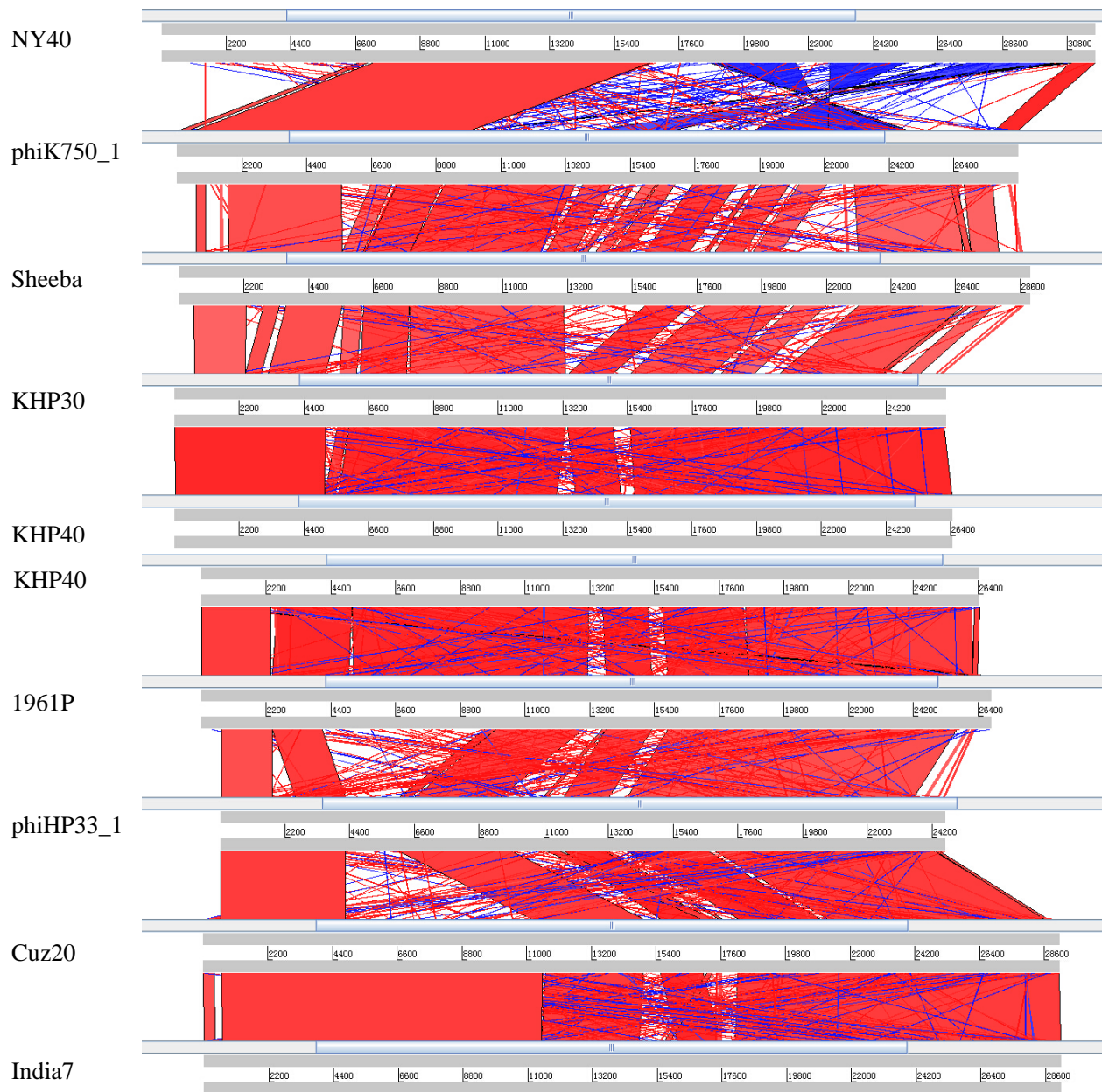

Figure S1. Comparative genomic analyses of clusterA1 phages of *Helicobacter*

Supplement: Figure S1 [file peerj-04-2012-s001.pdf]

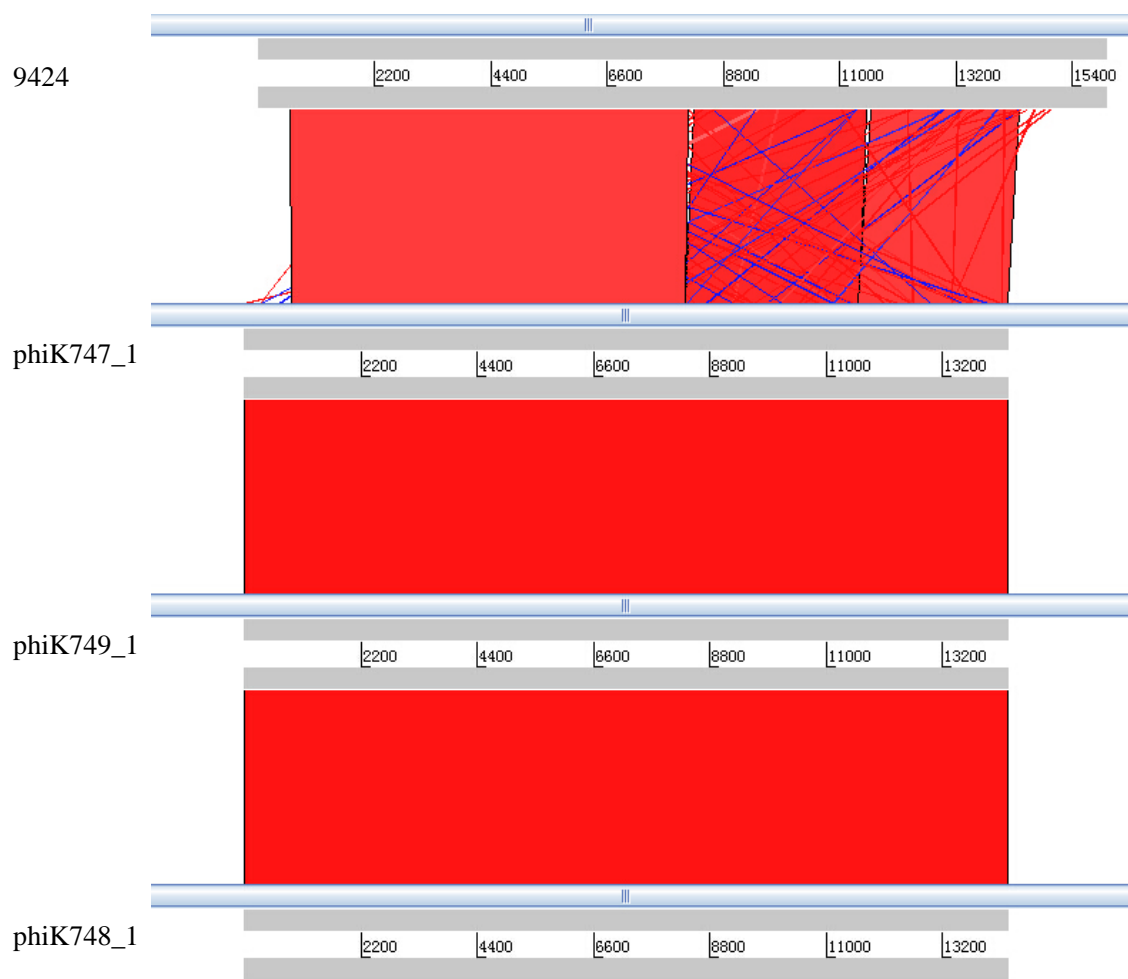

Figure S2. Comparative genomic analyses of clusterA2 phages of *Helicobacter*

Supplement: Figure S2 [file peerj-04-2012-s002.pdf]
