# Supplementary material for: Comparative analysis of prophage-like elements in Helicobacter sp. genomes
Source: PeerJ. 2016 May 5;4:e2012. doi: 10.7717/peerj.2012 (PMC4860318; doi:10.7717/peerj.2012)
Supplement: Table S1 [file peerj-04-2012-s003.doc]

Table S1 *Helicobacter* genomes retrieved in this study.

| Host | GenBank ID | Prophage |
| --- | --- | --- |
| *Helicobacter pylori* 26695 | AE000511.1 | - |
| *Helicobacter pylori* J99 | AE001439.1 | - |
| *Helicobacter pylori* 51 | CP000012.1 | - |
| *Helicobacter pylori* HPAG1 | CP000241.1 | - |
| *Helicobacter pylori* Shi470 | CP001072.2 | - |
| *Helicobacter pylori* G27 | CP001173.1 | - |
| *Helicobacter pylori* P12 | CP001217.1 | - |
| *Helicobacter pylori* 52 | CP001680.1 | - |
| *Helicobacter pylori* B38 | FM991728.1 | B38 |
| *Helicobacter pylori* v225d | CP001582.1 | - |
| *Helicobacter pylori* 908 | CP002184.1 | - |
| *Helicobacter pylori* SJM180 | CP002073.1 | - |
| *Helicobacter pylori* PeCan4 | CP002074.1 | - |
| *Helicobacter pylori* Cuz20 | CP002076.1 | Cuz20 |
| *Helicobacter pylori* Sat464 | CP002071.1 | - |
| *Helicobacter pylori* 35A | CP002096.1 | - |
| *Helicobacter pylori* India7 | CP002331.1 | India7 |
| *Helicobacter pylori* Gambia94/24 | CP002332.1 | Gambia94/24 |
| *Helicobacter pylori* Lithuania75 | CP002334.1 | - |
| *Helicobacter pylori* SouthAfrica7 | CP002336.1 | - |
| *Helicobacter pylori* 2017 | CP002571.1 | - |
| *Helicobacter pylori* 2018 | CP002572.1 | - |
| *Helicobacter pylori* B8 | FN598874.1 | - |
| *Helicobacter pylori* 83 | CP002605.1 | - |
| *Helicobacter pylori* Puno120 | CP002980.1 | - |
| *Helicobacter pylori* Puno135 | CP002982.1 | - |
| *Helicobacter pylori* SNT49 | CP002983.1 | - |
| *Helicobacter pylori* ELS37 | CP002953.1 | - |
| *Helicobacter pylori* HUP-B14 | CP003486.1 | - |
| *Helicobacter pylori* XZ274 | CP003419.1 | - |
| *Helicobacter pylori* F16 | AP011940.1 | - |
| *Helicobacter pylori* F30 | AP011941.1 | - |
| *Helicobacter pylori* F32 | AP011943.1 | - |
| *Helicobacter pylori* F57 | AP011945.1 | - |
| *Helicobacter pylori* Shi417 | CP003472.1 | - |
| *Helicobacter pylori* Shi169 | CP003473.1 | - |
| *Helicobacter pylori* Shi112 | CP003474.1 | - |
| *Helicobacter pylori* PeCan18 | CP003475.1 | - |
| *Helicobacter pylori* 26695 | CP003904.1 | - |
| *Helicobacter pylori* Rif1 | CP003905.1 | - |
| *Helicobacter pylori* Rif2 | CP003906.1 | - |
| *Helicobacter pylori* Aklavik117 | CP003483.1 | - |
| *Helicobacter pylori* Aklavik86 | CP003476.1 | - |
| *Helicobacter pylori* OK113 | AP012600.1 | - |
| *Helicobacter pylori* OK310 | AP012601.1 | - |
| *Helicobacter pylori* UM032 | CP005490.1 | phiK747_1 |
| *Helicobacter pylori* UM299 | CP005491.1 | phiK749_1 |
| *Helicobacter pylori* UM037 | CP005492.1 | phiK750_1 |
| *Helicobacter pylori* UM066 | CP005493.1 | - |
| *Helicobacter pylori* UM298 | CP006610.1 | phiK748_1 |
| *Helicobacter pylori* BM012A | CP006888.1 | - |
| *Helicobacter pylori* BM012S | CP006889.1 | - |
| *Helicobacter pylori* SouthAfrica20 | CP006691.1 | - |
| *Helicobacter pylori oki102* | CP006820.1 | - |
| *Helicobacter pylori oki112* | CP006821.1 | - |
| *Helicobacter pylori oki128* | CP006822.1 | - |
| *Helicobacter pylori oki154* | CP006823.1 | - |
| *Helicobacter pylori oki422* | CP006824.1 | - |
| *Helicobacter pylori oki673* | CP006825.1 | - |
| *Helicobacter pylori oki828* | CP006826.1 | - |
| *Helicobacter pylori oki898* | CP006827.1 | - |
| *Helicobacter pylori J166* | CP007603.1 | - |
| *Helicobacter pylori Strain: BM013A* | CP007604.1 | - |
| *Helicobacter pylori* *Strain: BM012B* | CP007605.1 | - |
| *Helicobacter pylori* *Strain: BM013B* | CP007606.1 | - |
| *Helicobacter pylori Strain: Hp238* | CP010013.1 | - |
| *Helicobacter pylori 26695-1* | CP010435.1 | - |
| *Helicobacter pylori 26695-1* *MET* | CP010436.1 | - |
| *Helicobacter pylori NY40* | AP014523.1 | phiNY40_1 |
| *Helicobacter pylori 26695-1* | AP013354.1 | - |
| *Helicobacter pylori 26695-1CL* | AP013356.1 | - |
| *Helicobacter pylori 26695-1CH* | AP013355.1 | - |
| *Helicobacter pylori J99* | CP011330.1 | - |
| *Helicobacter hepaticus ATCC 51449* | AE017125.1 | phiHH_1 |
| *Helicobacter acinonychis str.* Sheeba | AM260522.1 | Sheeba |
| *Helicobacter mustelae 12198* | FN555004.1 | - |
| *Helicobacter felis ATCC 49179* | FQ670179.2 | - |
| *Helicobacter cetorum* MIT 99-5656 | CP003481.1 | phiHCD_1 |
| *Helicobacter cinaedi ATCC BAA-847* | AP012492.1 | - |
| *Helicobacter bizzozeronii CIII-1* | FR871757.1 | phiHBZC1_1 |
| *Helicobacter canadensis MIT 98-5491* | CM000776.2 | - |

‘-’ means that prophage is not found.
