# Supplementary material for: Comparative analysis of prophage-like elements in Helicobacter sp. genomes
Source: PeerJ. 2016 May 5;4:e2012. doi: 10.7717/peerj.2012 (PMC4860318; doi:10.7717/peerj.2012)
Supplement: Table S2 [file peerj-04-2012-s004.doc]

Table S2. Genes of prophage phiHH_1.

| Gene | Function | Whether it is similar to phage protein |
| --- | --- | --- |
| HH_0750 | integrase | yes |
| HH_0751 | hypothetical protein | no |
| HH_0752 | DNA transposition protein | yes |
| HH_0753 | hypothetical protein | no |
| HH_0754 | host-nuclease inhibitor protein Gam | yes |
| HH_0755 | Rha family transcriptional regulator | yes |
| HH_0756 | DNA-binding protein rdgB | yes |
| HH_0757 | hypothetical protein | no |
| HH_0758 | hypothetical protein | no |
| HH_0759 | hypothetical protein | yes |
| HH_0760 | Ran GDP/GTP exchange factor | no |
| HH_0761 | Phage Tail Collar Domain family | yes |
| HH_0762 | DNA helicase | no |
| HH_0763 | DNA methyltransferase | yes |
| HH_0764 | hypothetical phage membrane protein | yes |
| HH_0765 | transporter | no |
| HH_0766 | hypothetical protein | no |
| HH_0767 | hypothetical protein | yes |
| HH_0768 | hypothetical protein | no |
| HH_0769 | hypothetical protein | no |
| HH_0770 | hypothetical protein | no |
| HH_0771 | putative tape measure protein | yes |
| HH_0772 | carbohydrate-binding protein | yes |
