# Supplementary material for: Comparative analysis of prophage-like elements in Helicobacter sp. genomes
Source: PeerJ. 2016 May 5;4:e2012. doi: 10.7717/peerj.2012 (PMC4860318; doi:10.7717/peerj.2012)
Supplement: Table S3 [file peerj-04-2012-s005.doc]

Table S3. Genes of prophage phiHCD_1.

| Gene | Function | Whether it is similar to phage protein |
| --- | --- | --- |
| HCD_00885 | thioredoxin | no |
| HCD_00890 | glycosyl transferase | no |
| HCD_00895 | ribonuclease | no |
| HCD_00900 | terminase | yes |
| HCD_00905 | hypothetical protein | yes |
| HCD_00910 | phage tail tape measure protein | yes |
| HCD_00915 | hypothetical protein | no |
| HCD_00920 | phage structure protein | yes |
| HCD_00925 | major capsid protein | yes |
| HCD_00930 | phage structure protein | yes |
| HCD_00935 | UV radiation resistance protein | yes |
| HCD_00940 | hypothetical protein | yes |
| HCD_00945 | phage prohead protease | yes |
| HCD_00950 | ribonuclease | no |
| HCD_00955 | phage tail protein | yes |
| HCD_00960 | phage structure protein | yes |
| HCD_00965 | hypothetical protein | no |
| HCD_00970 | hypothetical protein | yes |
| HCD_00975 | hypothetical protein | yes |
| HCD_00980 | hypothetical protein | no |
| HCD_00985 | hypothetical protein | no |
| HCD_00990 | holin | yes |
| HCD_00995 | aminodeoxychorismate lyase | no |
| HCD_01000 | phage hypothetical protein | no |
| HCD_01005 | hypothetical protein | yes |
| HCD_01010 | portal protein | yes |
| HCD_01015 | transposase | yes |
| HCD_01020 | transposase | yes |
