# Supplementary material for: Comparative analysis of prophage-like elements in Helicobacter sp. genomes
Source: PeerJ. 2016 May 5;4:e2012. doi: 10.7717/peerj.2012 (PMC4860318; doi:10.7717/peerj.2012)
Supplement: Table S4 [file peerj-04-2012-s006.doc]

Table S4. Genes of prophage phiHBZC_1.

| Gene | Function | Whether it is similar to phage protein |
| --- | --- | --- |
| HBZC1_17420 | phage DNA invertase | yes |
| HBZC1_17430 | transposase | yes |
| HBZC1_17440 | phage terminase large subunit | yes |
| HBZC1_17450 | hypothetical protein | no |
| HBZC1_17460 | phage late control D family protein | yes |
| HBZC1_17470 | phage tail protein | yes |
| HBZC1_17480 | phage tail tape measure protein | yes |
| HBZC1_17490 | phage tail tape measure protein | yes |
| HBZC1_17500 | phage tail tape measure protein | yes |
| HBZC1_17510 | hypothetical protein | no |
| HBZC1_17520 | hypothetical protein | no |
| HBZC1_17530 | phage tail protein | yes |
| HBZC1_17540 | phage tail protein | yes |
| HBZC1_17550 | phage tail protein | yes |
| HBZC1_17560 | phage tail sheath-like protein | yes |
| HBZC1_17570 | DNA methyltransferase | yes |
| HBZC1_17580 | cell division protein FtsB | no |
| HBZC1_17590 | hypothetical protein | no |
| HBZC1_17600 | holin | yes |
| HBZC1_17610 | hypothetical protein | yes |
| HBZC1_17620 | lysozyme protein | yes |
| HBZC1_17630 | phage tail protein | yes |
| HBZC1_17640 | phage tail protein | yes |
| HBZC1_17650 | methyl-accepting chemotaxis protein | no |
| HBZC1_17660 | tail protein | yes |
| HBZC1_17670 | phage baseplate protein | yes |
| HBZC1_17680 | type VI secretion protein | no |
| HBZC1_17690 | hypothetical protein | no |
| HBZC1_17700 | multidrug resistance protein D | no |
| HBZC1_17710 | DNA-methyltransferase | no |
| HBZC1_17720 | ABC transporter substrate-binding protein | no |
| HBZC1_17730 | spermidine synthase 2 | no |
| HBZC1_17740 | phage capsid protein | yes |
| HBZC1_17750 | phage capsid protein | yes |
| HBZC1_17760 | hypothetical protein | no |
| HBZC1_17770 | DNA adenine methylase | no |
| HBZC1_17780 | DNA adenine methylase | no |
| HBZC1_17790 | DNA primase | no |
| HBZC1_17800 | hypothetical protein | no |
| HBZC1_17810 | hypothetical protein | no |
| HBZC1_17820 | addiction module antitoxin RelB | no |
| HBZC1_17830 | DNA polymerase | yes |
| HBZC1_17840 | hypothetical protein | no |
| HBZC1_17850 | hypothetical protein | no |
| HBZC1_17860 | portal protein | yes |
| HBZC1_17870 | hypothetical protein | no |
| HBZC1_17880 | phage terminase large subunit | yes |
| HBZC1_17890 | ribonuclease | no |
| HBZC1_17900 | phage baseplate assembly protein V | yes |
| HBZC1_17910 | hypothetical protein | no |
| HBZC1_17920 | hypothetical protein | no |
| HBZC1_17930 | repressor LexA | yes |
| HBZC1_17940 | hypothetical protein | no |
| HBZC1_17950 | hypothetical protein | no |
| HBZC1_17960 | Replicative DNA helicase | no |
| HBZC1_17970 | YcfA family protein | yes |
| HBZC1_17980 | hypothetical protein | yes |
| HBZC1_17990 | site-specific recombinase phage integrase family | yes |
